# Xanthogranulomatous pyelonephritis infected with *the Providencia stuartii*: a case report and literature review

**DOI:** 10.1186/s12882-021-02565-x

**Published:** 2021-10-29

**Authors:** Zhunan Xu, Tong Cai, Xuebao Zhang, Jitao Wu, Chu Liu

**Affiliations:** 1 Binzhou Medical University, Yantai, Shandong China; 2 Department of Urology, The Affiliated Yantai Yuhuangding Hospital of Qingdao University, NO. 20 East Yuhuangding Road, Yantai, 264000 Shandong China

**Keywords:** Xanthogranulomatous pyelonephritis (XGP), Diagnosis, Treatment, Case report

## Abstract

**Background:**

Xanthogranulomatous pyelonephritis (XGP) is a rare and severe chronic inflammatory disease of the renal parenchyma, which is most commonly associated with super-infections by bacteria such as *E. coli*, *Proteus mirabilis*, and occasionally Pseudomonas species.

**Case presentation:**

Herein, we present a rare case of a patient with XGP infected with *Providencia stuartii*. Initially, the patient refused nephrectomy and underwent holmium laser lithotripsy and right ureteral stenting, followed by meropenem treatment of 7 days. Relapse occurred in the third month after discharge from the hospital, due to which she underwent a radical nephrectomy.

**Discussion:**

The diagnosis of XGP is confirmed by histopathology. The standard treatment for XGP is antibiotic therapy and radical nephrectomy, but partial nephrectomy may be appropriate in select cases.

## Background

Xanthogranulomatous pyelonephritis (XGP) is an unusual and severe chronic inflammatory disease of the renal parenchyma, which is characterized by infiltration of the renal parenchyma with lipid-laden macrophages [1]. The disease was first described in 1916 [2]. The cause of XGP remains unclear. However, obstruction of urinary flow and chronic bacterial infection are considered to be associated with XGP development [3]. XGP occurs approximately 1% of adults with pyelonephritis and 16% of pediatric nephrectomy cases [4, 5]. Typical clinical symptoms of XGP are fever of unknown origin, abdomen/flank pain, weight loss, anemia or palpable renal mass. Herein, we present a case of XGP infected with *Providencia stuartii*.

## Case presentation

A 32-year-old woman presented at our department with a 2-year history of right-side lower back paroxysmal pain and fever, which had worsened for 1 week. She had no history of the urinary tract infection, diabetes, hypertension or trauma. Physical examination revealed right renal percussive pain, with no positive signs on the left side.

Her laboratory findings were as follows: white blood cell (WBC) count = 32,000/ul; C-reactive protein = 20 mg/L (0–0.5 mg/dl) and procalcitonin = 0.52 ng/ml, (0–0.05 ng/ml). Urine analysis showed 4+ WBC. Urine culture was positive for *P. stuartii*, which was susceptible to meropenem and cefoxitin, and resistant to levofloxacin and ceftriaxone.

**Fig. 1:**
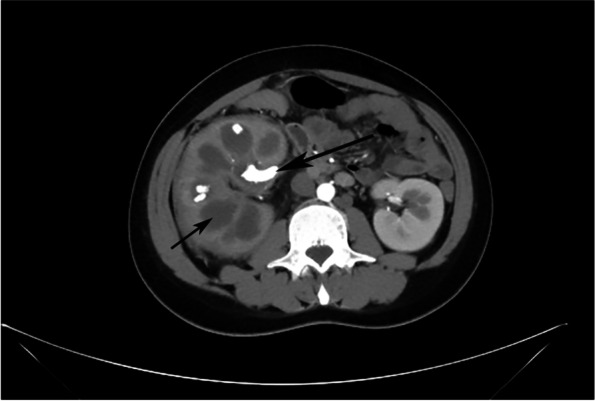
“Bear paw sign”: multiple, rounded, low density areas with enhancing rings arranged in a hydronephrotic pattern and hypoenhancement of the renal parenchyma (Small arrow). Multiple renal pelvis stones (Big arrow)

Urological ultrasonography (USG) showed that the right kidney was diffusely enlarged (127 × 74 mm). Several stones were detected within the right renal pelvis and calyceal groups. The largest one was about 20 mm × 9 mm. The left kidney was normal in size and echogenicity. Computerized tomography (CT) revealed multiple, rounded, low density areas with enhancing rings arranged in a hydronephrotic pattern and hypoenhancement of the renal parenchyma, right renal multiple pelvis stones and diffuse enlargement. The fat space around the right kidney was fuzzy, the right ureter was unevenly expanded, the internal density was increased, and the peripheral fat space was rough. The size and shape of the left kidney were normal, with no obvious abnormality. Multiple lymph nodes were found in the retroperitoneum, the diameter of the largest lymph node was about 12 mm (Fig. 1). Renal dynamic imaging revealed glomerular filtration rate (GFR) of right kidney = 17.17 ml/min, and left kidney = 83.74 ml/min.

**Fig. 2:**
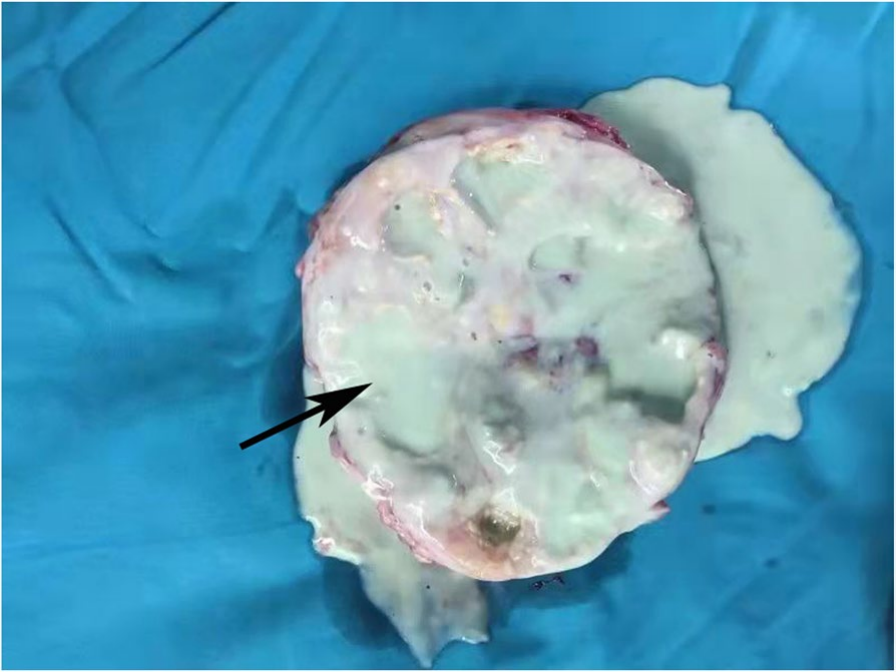
The right kidney: dilated calyces filled with pus

**Fig. 3:**
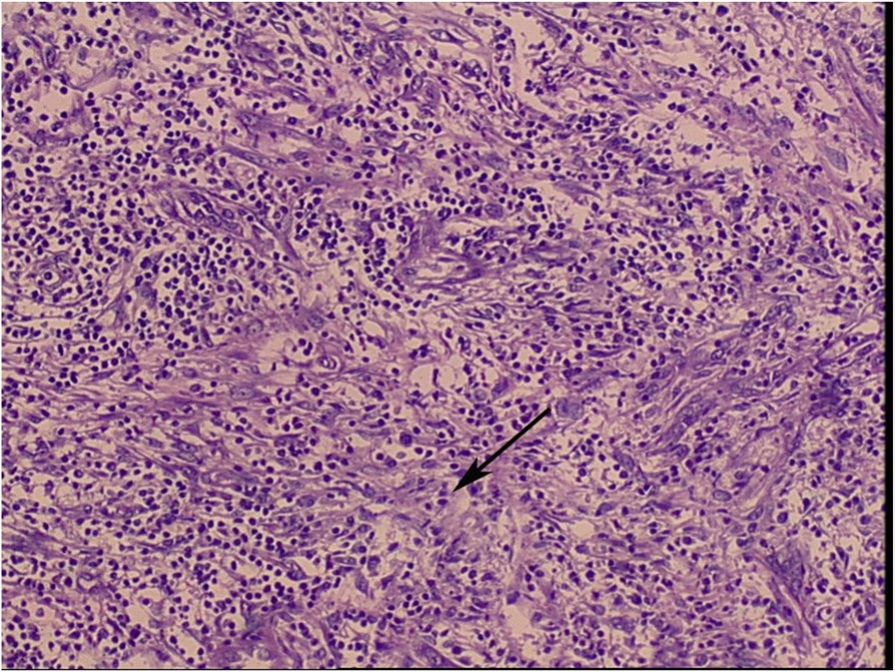
Pathology (HE× 4): diffuse inflammatory infiltrates of the renal medulla, and the black arrow points fat laden macrophage

Based on these findings, the patient was diagnosed with right XGP and right kidney stone. The patient refused nephrectomy, and underwent holmium laser lithotripsy and right ureteral stenting. She was given meropenem treatment after the lithotripsy, with complete explanation of the treatment. Seven days later, meropenem was replaced with cefoxitin. Two weeks after the lithotripsy, the patient’s symptoms disappeared and the patient requested to be discharged. However, she suffered right-side lower back paroxysmal pain and fever again in the third month after discharge from the hospital, due to which she underwent a radical nephrectomy. Initially, we attempted laparoscopic radical nephrectomy, but it was difficult to isolate the kidney due to serious perirenal fat adhesion and infiltration of blood during the operation, which led to conversion to open surgery. After surgery, we saw that the left kidney was enlarged, with intensely dilated calyces and filled with pus (Fig. 2). Postoperative pathology showed extensive glomerular fibrosis, significant hyperplasia of interstitial fibrous tissue with infiltration of numerous lymphocytes, plasma cells and foam cells, and focal abscess and necrotic lesions. Combined with medical history and histological morphology, it was consistent with inflammatory lesions (Fig. 3). She was given cefoxitin treatment after the nephrectomy for 7 days. The patient recovered well after 3 months of follow-up with regular Blood routine and CT of renal.

## Discussion and literature review

Xanthogranulomatous pyelonephritis (XGP) is an unusual and severe chronic inflammatory disease of the renal parenchyma, which is characterized by destruction of the renal parenchyma and granulomatous inflammation, with lipid-laden foamy macrophages as well as inflammatory infiltration and extensive renal fibrosis. Typical clinical symptoms of XGP are fever of unknown origin, abdomen/flank pain, weight loss, anemia or palpable renal mass. Korkes F and colleagues retrospectively reviewed 41 cases of XGP, and all patients were found to be symptomatic [6]. XGP may occur in all age groups, but it predominantly affects adults and is sporadically diagnosed in children [1]. Gender may be related to typing of XGP. Some reports have indicated that the diffuse form is equally observed in boys and girls, while focal XGP is more common in girls [7–9]. XGP often occurs unilaterally, and bilateral cases are extremely rare. The etiology of XGP remains unclear. Recurrent urinary tract infections (54%), obstructive nephropathy (68%), malnutrition, abnormal lipid metabolism, altered immunological response (5%), lymphatic blockage, and congenital urinary anomalies (5%) have been reported to predispose an individual to this rare renal parenchymal infection often mimicking neoplastic renal disorder [1, 10]. The most common pathogenic bacteria were *Escherichia coli* (30%), Klebsiella (19%), Proteus (8%), Pseudomonas (5%), Enterococcus (5%) and Candida spp. (5%) [11].

XGP has been termed “the great imitator” because the differential diagnosis includes a large group of diseases such as Wilms tumor, renal cell carcinoma, renal abscess, infected renal cystic disease, tuberculosis, malakoplakia, and transitional renal cell carcinoma. Computerized tomography (CT) scan is critical for preoperative evaluation of XGP, while ultrasonography and magnetic resonance imaging can also be used. Based on CT findings, XGP can be divided into diffuse type (92%) or focal type (8%) [12, 13]. Typical CT features of diffuse XGP are destruction of renal parenchyma, which is replaced by multiple, rounded, low density areas with enhancing rings arranged in a hydronephrotic pattern and hypoenhancement of the renal parenchyma, described as “bear paw sign”. The CT image of our patient also showed typical “bear paw sign”. In focal XGP patients, CT frequently shows a well-defined localized intra-renal lesion with hypo-attenuation. Depending on the extent of inflammation, XGP can be classified as three stages: nephric XGP (stage I: The lesion is confined to the renal parenchyma.), perinephric XGP (stage II: The lesion penetrates the renal parenchyma and invade the perirenal fat.) and paranephric XGP (stage III: The lesion pervades most or all of the kidneys and extensively involves perirenal tissue and the posterior peritoneu.) [14]. If the clinical and imaging features are nonspecific along with laboratory findings, XGP is confirmed by histopathology [15]. Preoperative renal mass biopsy is the gold standard to avoid misdiagnosis and mistreatment, although false negative results were possible [16].

The standard curative treatment for diffuse XGP is antibiotic therapy and nephrectomy but focal XGP can be treated with antibiotics or nephron-sparing surgery. Korkes F et al. reported 41 cases of XGP, and all patients underwent nephrectomy. Except for two died from septic shock after surgery, the rest of the patients recovered well [6]. Çaliskan S et al. reported 13 cases of XGP, of which one patient underwent partial nephrectomy and 12 patients underwent nephrectomy. Perioperative and postoperative complications did not occur [17]. Chlif et al. reported a series where they treated three patients with localized XGP with partial nephrectomy. After a mean follow-up of 34.6 months, no recurrence of XGPN has been noted [12]. In our case, the patient had diffuse pyelonephritis, and the initial treatment with holmium laser lithotripsy and antibiotics failed, and nephrectomy was finally performed.

## Conclusion

XGP is a rare chronic inflammatory disease. A preoperative biopsy or intraoperative frozen section may be beneficial to confirm the diagnosis in suspected cases. Although the accepted curative treatment for XGP is antibiotic therapy and nephrectomy, partial nephrectomy may be appropriate in select cases. Different treatments can be chosen according to the type of XGP.

## Data Availability

Not applicable.

## Abbreviations

XGP: Xanthogranulomatous pyelonephritis
WBC: White blood cell
USG: Urological ultrasonography
CTU: Computerized tomography urography
CTA: Computerized tomography angiography
GFR: Glomerular filtration rate
CT: Computerized tomography

## References

[1] Sangüesa Nebot C, Picó Aliaga S, Serrano Durbá A, Roca MJ (2018). Xantogranulomatous pyeloneprhritis in children. Insights Imaging.

[2] Schlagenhaufer F (1916). Uber eigentumliche staphylomykosen der Nieren und des pararenalen Bindegewebes. Frankf Zt Pathol.

[3] Zhou G, Hu W, Bao H, Zhang Q (2015). A rare case of xanthogranulomatous pyelonepheritis with hepatic angiomyolipoma. Int J Clin Exp Pathol.

[4] Siddappa S, Ramprasad K, Muddegowda MK (2011). Xanthogranulomatous pyelonephritis: a retrospective review of 16 cases. Korean J Urol.

[5] Rao AG, Eberts PT (2011). Xanthogranulomatous pyelonephritis: an uncommon pediatric renal mass. Pediatr Radiol.

[6] Korkes F, Favoretto RL, Bróglio M, Silva CA, Castro MG, Perez MD (2008). Xanthogranulomatous pyelonephritis: clinical experience with 41 cases. Urology..

[7] Braun G, Moussali L, Balanzar JL (1985). Xanthogranulomatous pyelonephritis in children. J Urol.

[8] Clapton WK, Boucaut HA, Dewan PA, Bourne AJ, Byard RW (1993). Clinicopathological features of xanthogranulomatous pyelonephritis in infancy. Pathology..

[9] Hammadeh MY, Nicholls G, Calder CJ, Buick RG, Gornall P, Corkery JJ (1994). Xanthogranulomatous pyelonephritis in childhood: pre-operative diagnosis is possible. Br J Urol.

[10] Samuel M, Duffy P, Capps S, Mouriquand P, Williams D, Ransley P (2001). Xanthogranulomatous pyelonephritis in childhood. J Pediatr Surg.

[11] Chlif M, Chakroun M, Ben Rhouma S (2016). Xanthogranulomatous pyelonephritis presenting as a pseudotumour. Can Urol Assoc J.

[12] Ng CK, Yip SK, Sim LS (2002). Outcome of percutaneous nephrostomy for the management of pyonephrosis. Asian J Surg.

[13] Shah K, Parikh M, Gharia P, Modi PR (2012). Xanthogranulomatous pyelonephritis-mimicking renal mass in 5-month-old child. Urology..

[14] Hendrickson RJ, Lutfiyya WL, Karrer FM, Furness PD, Mengshol S, Bensard DD (2006). Xanthogranulomatous pyelonephritis. J Pediatr Surg.

[15] Cao D, Liu L, Gao L, Wei Q (2014). Ureteral calculi combined with xanthogranulomatous pyelonephritis mimicking renal tuberculosis in a male child. Kaohsiung J Med Sci.

[16] Posielski NM, Bui A, Wells SA (2019). Risk factors for complications and nondiagnostic results following 1,155 consecutive percutaneous Core renal mass biopsies. J Urol.

[17] Çaliskan S, Özsoy E, Kaba S, Koca O, Öztürk MI (2016). Xanthogranulomatous pyelonephritis. Arch Iran Med.

